# Early clinical and microbiological predictors of outcome in hospitalized patients with cryptococcal meningitis

**DOI:** 10.1186/s12879-022-07118-7

**Published:** 2022-02-09

**Authors:** Lidiane de Oliveira, Marcia de Souza Carvalho Melhem, Renata Buccheri, Oscar José Chagas, José Ernesto Vidal, Fredi Alexander Diaz-Quijano

**Affiliations:** 1 Department of Epidemiology, School of Public Health, University of São Paulo, Av. Dr. Arnaldo, 715, São Paulo, SP CEP 01246-904 Brazil; 2 Mycology Unit of Adolfo Lutz Institute, Public Health Reference Laboratory, Secretary of Health, Av. Dr.Arnaldo, 351, São Paulo, SP CEP 05411-000 Brazil; 3 School of Medicine, Federal University of Mato Grosso do Sul, Bairro Universitário, Av. Costa e Silva, s/no, Campo Grande, MS CEP 79070-900 Brazil; 4 Department of Neurology, Emílio Ribas Institute of Infectious Diseases, Av. Dr. Arnaldo 165, São Paulo, SP CEP 05411-000 Brazil; 5 Department of Infectious Diseases, Hospital das Clinicas, School of Medicine, University of São Paulo, Av. Dr. Enéas de Carvalho Aguiar, 470, São Paulo, SP CEP 01246-904 Brazil

**Keywords:** *Cryptococcus*, Cryptococcal meningitis, Predictors, Prognosis, Mortality

## Abstract

**Background:**

Cryptococcal meningitis causes high mortality in immunocompromised and immunocompetent patients. The objective of this study was to identify early predictors of clinical outcome, available at the first days of hospitalization, in patients with cryptococcal meningitis in a tertiary center in Brazil.

**Methods:**

Ninety-six cases of cryptococcal meningitis with clinical, epidemiological and laboratory data, and identification and antifungal susceptibility of the strains were analyzed. Quantitative CSF yeast counts were performed by direct microscopic exam with a Fuchs-Rosenthal cell counting chamber using an institutional protocol. Univariable and multiple analyses using logistic regression were performed to identify predictors, available at the beginning of hospitalization, of in-hospital mortality. Moreover, we performed a secondary analysis for a composite outcome defined by hospital mortality and intensive care unit transfer.

**Results:**

The species and the antifungal susceptibility were not associated with the outcomes evaluated. The variables significantly associated with the mortality were age (OR = 1.08, 95% CI 1.02–1.15), the cerebrospinal fluid (CSF) yeasts count (OR = 1.65, 95% CI 1.20–2.27), systemic arterial hypertension (OR = 22.63, 95% CI 1.64–312.91) and neurological impairment identified by computed tomography (OR = 41.73, 95% CI 3.10–561.65). At the secondary analysis, CSF yeast count was also associated with the composite outcome, in addition to the culture of *Cryptococcus* spp. from bloodstream and cerebral toxoplasmosis. The associations were consistent with survival models evaluated.

**Conclusions:**

Age and CSF yeast count were independently associated with in-hospital mortality of patients with cryptococcal meningitis but *Cryptococcus* species identification and antifungal susceptibility were not associated with the outcomes. Quantitative CSF yeast counts used in this study can be evaluated and implemented in other low and middle-income settings.

**Supplementary Information:**

The online version contains supplementary material available at 10.1186/s12879-022-07118-7.

## Background

Cryptococcosis is one of the major invasive diseases in humans. It is acquired by inhalation of fungal propagules, which can be deposited in the pulmonary alveoli to disseminate to cutaneous tissue, internal organs, and to the central nervous system, where is observed the most severe clinical form [1].

In people living with HIV/AIDS (PLWHA), cryptococcosis is one of the opportunistic AIDS-defining infections and one of the main causes of death [2–7].

The major agents of cryptococcosis are members of *C. neoformans* and *C. gattii* complex. This complex was recognized in 2015 into seven species, based on phenotypic and genotypic, geographic, epidemiological and virulence characteristics [8–15]. The new classification includes the species *C. neoformans* sensu strictu (s.s) (genotype VNI/VNII/VNB), hybrids between *C. neoformans* and *C. deneoformans* (genotype VNIII) and *C. deneoformans* (genotype VNIV), and *C. gattii* complex is formed by *C. gattii* s.s (VGI), *C. deuterogattii* (VGII), *C. bacillisporus* (VGIII), *C. tetragattii* (VGIV), and *C. decagattii* (VGIIIc/VGIV) [8, 16].

The major specie associated with immunocompromised remains *C. neoformans s.s.*, mainly in PLWHA, followed by *C. deneoformans,* C. *bacillisporus, C*. *tetragattii* and *C. decagattii.* On the other hand, *C. gattii and C*. *deuterogattii* are known to affect apparently immunocompetent individuals due to increased virulence and less antifungal susceptibility [16–19].

The lethality rates vary and depend on several factors, among them: underlying disease, stage of diagnosis of the disease and therapeutic strategy. However, it remains the second most common cause of AIDS-related mortality, resulting in the death of an average of 15% of this population [20]. In developed countries, cryptococcal meningitis presents a lethality range of 9% to 20%, and this rate in developing countries is around 40% [7, 21–24].

The antifungal therapy depends on the immunity of the patient, occurrence of underlying disease, site of infection, toxicity and availability of the antifungal drugs [7, 25, 26]. The recommended treatment for patients with HIV is divided into phases of induction, consolidation and maintenance. The preferred induction regimen recommended by the World Health Organization consists in a short-course (one-week) with amphotericin B deoxycholate (AMBd) and 5-flucytosine (5FC), followed by one week of fluconazole (FCZ). In contrast, the preferred induction regimen recommended by the Infectious Disease Society of America consists in at least two weeks with lipidic formulations of amphotericin B and 5-flucytosine (5FC) [7, 25, 26]. However, lipidic formulations of amphotericin B and 5FC are usually unavailable in public health services of Latin America.

Despite the recommendation of some experts to the antifungal susceptibility determination by minimum inhibitory concentration (MIC) in the management of cryptococcal meningitis, the prognostic value of this test has not been defined and the correlation between the susceptibility results and the clinical outcomes remains uncertain [27–29]. Another limitation is about fungicidal drugs such as AMB. The reference test, the microdilution, determines de minimum inhibitory concentration of the antifungal, and it may not be appropriate since it determines the inhibitory activity and thus is unable to identify resistant strains [3, 30–32].

The level of heteroresistance to FCZ (LHF) refers to the ability of cells to grow at high concentrations of the drug. These cells can generate homogenous populations, or clones, able of adapting to higher concentrations [33–35]. The occurrence of resistant strains during therapy, therapeutic failure and relapses can be attributed to LHF, mainly due to FCZ exposure at the maintenance phase [33, 36–38]. Time-Kill (TK) method is capable of indicating the kill of the strains according to the time of exposure to the drug. In addition, the maximum concentration of AMB-d available in serum is 1 mg/L [39, 40]. This method can be promising because of reports that presented a correlation of TK with outcomes of the patients with cryptococcosis [41, 42].

Considering all these developments in diagnostic tests, it is necessary to estabilish which are useful tools to predict the prognosis and guide therapeutic alternatives. There are few data about the potential association between *Cryptococcus* species, antifungal susceptibility, fungal burden and clinical outcomes of cryptococcal meningitis in Brazil. Consequently, the purpose of this study was to identify predictors of clinical outcome in patients hospitalized with cryptococcal meningitis in a tertiary center of São Paulo, Brazil.

## Methods

### Study design and population

We developed a retrospective cohort study, including patients in their first episode of cryptococcal meningitis, admitted at the Emílio Ribas Institute of Infectious Diseases (ERIID) of São Paulo from August 2012 to December 2013 and January 2015 to August 2017. For administrative reasons, episodes from 2014 could not be evaluated for this study.

The ERIID is a public tertiary hospital and the main reference for the treatment of infectious diseases in the state of São Paulo, Brazil.

### Data collection and clinical information

Demographic, clinical, and laboratory data were obtained from medical records. The variables evaluated included sex, age, duration of hospital stay, Glasgow coma scale, signs and symptoms of cryptococcosis at admission, antifungal therapy during the induction phase, information about HIV infection, viral load, CD4 values and occurrence of other infectious diseases or comorbidities. The antiretroviral therapy status was defined as antiretroviral naïve, regular, irregular and abandoned therapy.

The results of laboratory tests of cerebrospinal fluid (CSF) used were: opening pressure during the lumbar puncture, glucose and protein dosage, WBC count and differential count, *Cryptococcus* spp. antigen, culture, India ink test and quantitative CSF yeast cell counts using a validated protocol at ERIID [43]. This methodology is performed by direct microscopic exam with a Fuchs-Rosenthal cell counting chamber, quantifying how many yeast cells/mcL of CSF were present. Results of blood urea and creatinine level were also collected. All these variables were referred to the hospital admission. It was recorded results of brain computerized tomography (CT) and brain magnetic resonance imaging (MRI).

### Identification and antifungal susceptibility profile of strains

The first strain obtained from each case was sent and identified in the Mycology Unit of Adolfo Lutz Institute of São Paulo, using classic methods [44]. All strains were genotyped by Polymerase Chain Reaction-Restriction Fragment Length Polymorphism (PCR–RFLP) with the *URA5* gene amplification and treatment with enzymes Sau96I and HhaI [45]. The strains band profiles obtained by electrophoresis were plotted and compared with the profiles from *C. neoformans* reference strains: VNI-WM 148, VNII-WM 626, VNIII-WM 628 and VNIV-WM 629 and *C. gattii*: VGI- WM 179, VGII-WM 178, VGIII-WM 161 and VGIV-WM 779, provided by Oswaldo Cruz Foundation (FIOCRUZ) in Brazil.

The minimum inhibitory concentration (MIC) was determined by the broth microdilution described in E. Def. 7.3.1 of AFST-EUCAST [46] against FCZ and Etest® strips (bioMérieux, France) for AMB, following manufacturer's instructions. To interpret the results, breakpoints adopted in previous studies and clinically relevant were used: MIC < 8 mg/L and resistant MIC ≥ 16 mg/L for FCZ and MIC < 1 mg/L and resistant MIC > 2 mg/L for AMB [28, 29, 47]. The quality control strains *C. krusei (Pichia kudriavzevii)* ATCC 6258 and *C. parapsilosis* ATCC 22019 and the *C. neoformans* H99 and *C. deuterogattii* R265 were used in all tests.

The test to determine the LHF was performed in duplicate according to the method described in literature [34]. The strains were cultivated by spot test in plates of YPD agar with FCZ concentrations of 0, 8, 16, 32, 64, 128, and 256 mg/L and incubated at 30 °C for 72 h. The LHF was determined by the growth colonies on the plate with the highest FCZ concentration. These colonies, or clones, were cultivated in YPD agar plates with the FCZ concentration equivalent to the LHF.

The TK method was performed to determine the time to fungicide activity of 1 mg/L of AMBd [3, 41, 48]. The strains were cultivated in tubes with RPMI medium and the concentration of 1 mg/L of AMBd. These tubes were incubated at 30 °C and aliquots were diluted and cultivated in Sabouraud plates. This procedure was realized at time 0 (T0), as it refers to the moment without incubation of the cells with AMBd and repeated to 6 h later, i.e., T6, at T12, T24, T48 and T72. After 72 h of incubation, it was observed which the period of incubation with AMB was able to inhibit 99.9% of the UFC/mL, compared to the T0. The time required for this reduction was the endpoint of the test, the TK.

### Statistical analysis

The objective of this analysis was to identify predictors of prognosis. In that sense we considered as primary outcome in-hospital mortality. Moreover as a secondary analysis we explored additionally a composite outcome defined by the occurrence of in-hospital mortality and intensive care unit (ICU) transfer during hospitalization. The univariable analysis of categorical and quantitative variables was performed using Fisher's Exact Test and Wilcoxon-Mann–Whitney Test, respectively, and that presented a p-value ≤ 0.2 were selected to the logistic regression.

To avoid collinearity between the quantitative variables, the Pearson correlation coefficient was calculated and, among variables strongly correlated, the one with the highest association with the outcome was chosen. Consequently, the Glasgow scale was excluded from subsequent analysis since it was strongly correlated with the age (r = − 0.73) and presented lower association with mortality (age: OR = 1.06, 95% CI 1.02–1,11, *p* = 0.006; Glasgow scale OR = 0.59, 95% CI 0.35–0.99, *p* = 0.047).

Quantitative variables were analyzed regarding a possible linear relation with the outcome. Thus, the possibility of applying a transformation or defining cutoff points was explored. For this, the variables were categorized into deciles and then evaluated to about the odds of mortality.

The variables were evaluated by the logistic regression, and we obtained a multiple model only including those significantly associated with the outcome (p ≤ 0.05). The antifungal susceptibility profile and genotypes of the strains and the antifungal therapy were also evaluated in the multiple analysis, independently of the univariable results.

The goodness of fit was evaluated by the Pearson test and the Hosmer–Lemeshow test. The predicted values were used to elaborate the receiver operating characteristic (ROC) curves, and the accuracy of the model was calculated for the cutoff of 0.5. It was evaluated whether the associations were maintained by excluding the outliers (upper values three times the average of the leverage) and influential observations (with delta-deviance higher than the cutoff point of 4) [49].

Cox regression was performed to analyze whether these predictors were associated with time to the outcome and the Kaplan–Meier curves were obtained to represent graphically the survival time from the date of hospitalization to the occurrence of the outcomes. For this analysis, the patients who survived were censored on the day of hospital discharge, and for the secondary analysis, the patients who did not  develop the corresponding outcome were censored on the day of hospital discharge.

All analyses were performed using the Stata® program (version 11.0, Stata Corp. LP, College Station, TX, USA).

## Results

### Clinical and laboratory information

We analyzed 96 cases of cryptococcal meningitis in patients aged 19 to 68 years old and the majority were male (74/96; 77.08%). The duration of hospital stay ranged from 1 to 202 days with a median of 38 days and an interquartile range (IQR) of 23–54, and ICU transfer occurred in 27 (30%) patients, with a stay period of 1 to 48 days and median of seven days in this unit. Hospital death occurred in 25 patients (25/93, 26.9%) who remained hospitalized between 1 and 202 days with a median of 23 days until the death. Relapse in up to 6 months was reported in 25.8% (16/62) of survival.

HIV-infection was described at 96.7% (87/90) of patients with viral load from undetectable (< 50 copies/mL), in eight of them, to 3.436.979 copies/mL (median 104.126, IQR 1824–332.156 copies/mL). CD4 values ranged from 2 to 722 cells/mm^3^ (median 42, IQR 16–78). Moreover, one or more comorbidities were recorded in 27 (30%) patients, being the most frequent: viral hepatitis infection (11/90, 12.2%), Kaposi's sarcoma (7/90; 7.8%) and systemic arterial hypertension (diastolic blood pressure equal to or above 130 mmHg by 80 mmHg [50]) (6/90, 6.7%). Other infections were diagnosed in 58.8% (53/90) of patients and the most frequently were oral/esophageal candidiasis (20; 22.1%), pulmonary tuberculosis (15; 16.7%), cytomegalovirus infection (12; 13.3%), bacterial pneumonia (11; 12.2%), and cerebral toxoplasmosis (9; 10%). About the cases with tuberculosis diagnosis, 2 cases presented pulmonary and meningeal co-infection. It was observed less than 10% of cases of syphilis, pneumocystosis and tuberculous meningitis. Regular antiretroviral therapy was recorded for 19.5% (17/87) of HIV-infected patients, 33.3% (29/87) cases were antiretroviral naive, 26.4% (23/87) related irregular treatment and 19.5% (17/87) abandoned the therapy. Combination antifungal therapy AMBd plus FCZ (AMBd 0.7–1 mg/kg/day + FCZ 800–1200 mg/day) was used in 47.2% (42/89) of patients and AMBd plus 5FC (AMBd 0.7–1 mg/kg/day + 5FC 100 mg/kg/day) was prescribed to 43.8% (39/89) of patients. Combination of AMBd plus FCZ plus 5FC (AMBd 0.7–1 mg/kg/day + FCZ 800–1200 mg/day + 5FC 100 mg/kg/day) was prescribed to 6.7% (6/89) patients. AMBd monotherapy was prescribed in only two (2.2%) patients.

The CSF opening pressure was recorded in 69 (76.7%) patients and the value ranged from 8 to 150 cmH_2_O (median 33, IQR 22.4–50 cm H_2_O). Intracranial hypertension (CSF opening pressure > 20 cm H_2_O) was observed in 54 (78.6%) patients. The CSF WBC count presented values from 0 to 800 cells/mL (median 55, IQR 3–55 cells/mL). The CSF yeast count presented values ranging from 0 to 11.520 yeasts/mm^3^ (median 192 yeasts/mm^3^). Cultures of *Cryptococcus* spp. from extra neural sites were observed in 20 (22.5%) patients, including 18 from the bloodstream, three from the skin, one from lymph nodes and one from lungs. All cases were isolated *C. neoformans s.s.*

Abnormalities in neuroimaging were observed in 50 patients. According to MRI results, the most observed alterations were the presence of pseudocysts and cryptococcomas with 5 (10%) occurrences from each. The most observed alterations by CT were cortical atrophy (15; 30%), cortical edema and ventricular dilatation (4; 8% each).

### Identification and antifungal susceptibility of strains

It was identified 76 (81.7%) *C. neoformans* VNI, 17 (18.3%) *C. neoformans* VNII and three strains of *C. deuterogattii *(3.2%). The AMBd-MIC varied from 0.012 mg/mL to 0.94 mg/L, and resistance were not observed. The FCZ-MIC was 0.12 mg/L to 64 mg/L and 28.1% of strains (27/96) were FCZ-resistant (MIC ≥ 16 mg/L). LHF values ranged from 16 to 128 mg/L, in which 9.4% of strains with LHF 16 mg/L (9/96), 53.1% with 32 mg/L (51/96), 32.3% with 64 mg/L (31/96) and 5,2% (5/96) with LHF 128 mg/L. Fungicidal activity of AMBd at TK6 was observed to 20.8% (20/96), at TK12 to 19.8% (19/96), at TK24 to 23.9% (23/96), at TK48 to 17.7% (17/96) and at TK72 to 11.5% (11/96) of the strains. No fungicidal activity was observed in 6.2% of the strains (6/96).

### Predictors of mortality

In the univariable analysis, the following variables were associated with in-hospital mortality: age, Glasgow coma scale, ICU transfer, torpor, systemic arterial hypertension, pneumonia, the culture of *Cryptococcus* spp. from extra neural sites, CSF yeasts count and urea level, and presence of cerebral edema and ventricular dilation by CT (Table 1).

**Table 1:** Univariable analysis of prognostic variables related to hospital mortality in 96 patients with cryptococcal meningitis at Emilio Ribas Institute of Infectious Diseases, São Paulo, Brazil

Variables	All	Deaths	Survivors	p^*^
Age (years), average (IQR)	38.7 (29–46)	44.2 (36–52.5)	36.4 (28–44.5)	0.007
Glasgow coma scale, average (IQR)	15 (15–15)	14 (14–15)	15 (15–15)	< 0.001
ICU transfer, n (%)	27/90 (30%)	15/22 (68.2%)	12/68 (17.7%)	< 0.001
Signs and symptoms at admission				
Nausea	28/90 (31.1%)	6/22 (27.5%)	22/68 (32.4%)	0.07
Seizures	12/90 (13.3%)	6/22 (27.5%)	6/68 (8.8%)	0.06
Mental confusion	19/90 (21.1%)	8/22 (36.4%)	11/68 (16.2%)	0.07
Torpor	4/90 (4.4%)	3/22 (13.6%)	1/68 (1.5%)	0.004
Clinical-laboratory data				
Systemic arterial hypertension	6/90 (6.7%)	5/22 (22.7%)	1/68 (1.5%)	0.003
Pneumonia	11/90 (12.2%)	6/22 (27.3%)	5/68 (7.4%)	0.02
Culture of *Cryptococcus spp.* from extra neural sites	20/89 (22.5%)	9/22 (40.9%)	11/67 (16.4%)	0.04
Culture of *Cryptococcus* spp. from bloodstream	18/89 (20.2%)	8/22 (36.4%)	10/67 (14.9%)	0.06
Culture of *Cryptococcus* spp. from skin	3/89 (3.4%)	2/22 (9.1%)	1/67 (1.5%)	0.15
CSF opening pressure (cmH_2_O)-average (IQR)	39.4 (22.4–50)	47.9 (26.2–60.5)	36.8 (20.5–50)	0.20
CSF yeasts cout (yeasts/mm^3^)-average (IQR)	749 (6–665)	1696 (340–2240)	443.2 (3–384)	< 0.001
Urea level (mg/dL)-average (IQR)	33.41 (21–40)	42.9 (22–48)	30.0 (21–35)	0.06
Time-kill of 1 mg/L AMB (hours)-average (IQR)	27.9 (12–48)	24 (6–24)	29.8 (12–48)	0.17
Neuroimage-computed tomography				
Cerebral edema	4/90 (4.4%)	3/22 (13.6%)	1/68 (1.5%)	0.04
Ventricular dilation	4/90 (4.4%)	3/22 (13.6%)	1/68 (1.5%)	0.04

^*^p ≤ 0.2; IQR, interquartile range 25–75%; CSF, cerebrospinal fluid; AMB, amphotericin B; ICU, intensive care unit

**Fig. 1:**
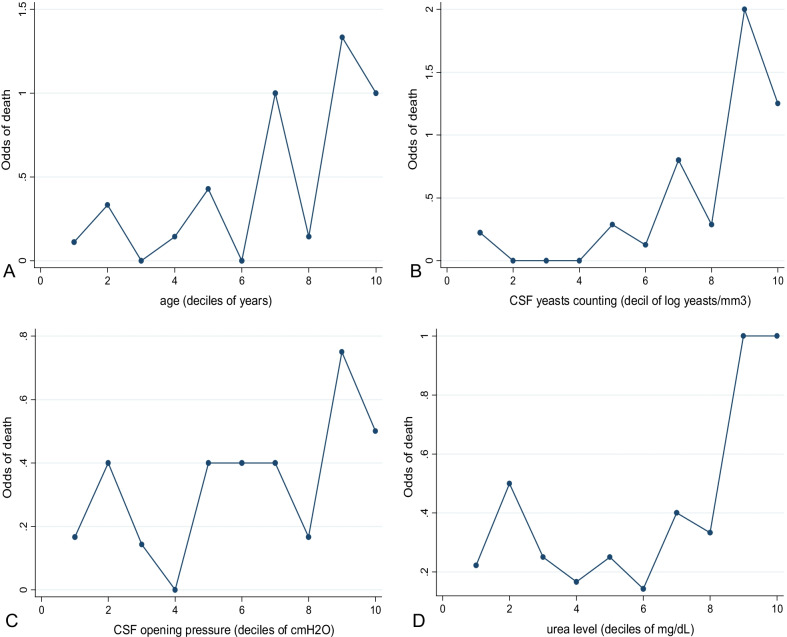
Graphs of the quantitative variables associated with mortality, transformed into deciles **A** Age, **B** CSF yeast count, **C** CSF opening pressure, and **D** Blood urea level

We observed trends compatible with a linear shape when odds of mortality were plotted regarding the deciles of age, CSF yeasts count and CSF opening pressure (Fig. 1A–C respectively). The variable urea level showed stabilization of odds at 40 mg/dL. For this reason, it was possible to categorize it (deciles nine and ten at Fig. 1D).

The CSF yeast count was transformed to a logarithm version. However, a unit was added to all observations before transforming into logarithm, to avoid losses related to the natural logarithm of 0. This variable, hereafter named as “CSF yeast count-log”, had a higher association with the outcome than the original version.

The variables cerebral edema and ventricular dilation by CT were unified, due to the low number of observations. This variable was defined by “neurological impairment identified by computed tomography”.

The variables associated with hospital mortality in the multiple analysis were: age, CSF yeast count-log, systemic arterial hypertension and neurological impairment by computed tomography (Table 2). The antifungal susceptibility profile, the genotypes strains, antifungal therapy regimen and antiretroviral therapy were not associated with mortality.

**Table 2:** Multiple analysis by logistic regression of variables associated with hospital mortality in 96 patients with cryptococcal meningitis at Emilio Ribas Institute of Infectious Diseases, São Paulo, Brazil

Variable	Odds ratio	Confidence interval 95%	*p*
Age (years)	1.08	1.02–1.15	0.007
CSF yeast count-log	1.65	1.20–2.27	0.002
Systemic arterial hypertension	22.63	1.64–312.91	0.02
Neurological impairment by computed tomography	41.73	3.10–561.65	0.005

CSF, cerebrospinal fluid

**Fig. 2:**
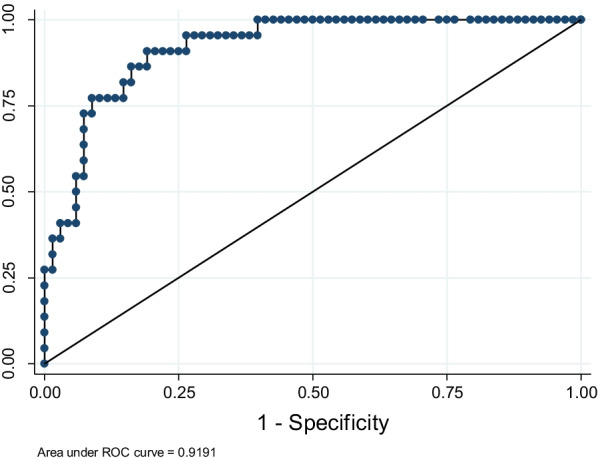
ROC curve using the variables age in years, CSF yeast count-log, systemic arterial hypertension and neurological impairment by computed tomography associated with hospital mortality

The model showed an area under the ROC curve of 91.9% (95% Confidence Interval [95% CI] 0.86–0.97%, Fig. 2) and presented an accuracy of 83.3%. The sensitivity and specificity were 54.5% and 92.6%, respectively (positive predictive value = 70.6%, negative predictive value = 86.6%). The model presented goodness of fit at the Pearson test and the Hosmer–Lemeshow test with *p* = 0.95 and 0.99, respectively.

By excluding the extreme and influential observations, the associations with the outcome were maintained (see Additional file 1: Fig. S1, Tables S1 and S2).

The categorical variable of urea level was associated with the outcome (Odds Ratio [OR] = 6.16, 95% CI 1.31–29.00, *p* = 0.02), however, its inclusion resulted in the loss of precision due to the increase at the standard error of other estimates, such as systemic arterial hypertension (95% CI increased to 2.655–26.91) and neurological impairment by computed tomography (95% CI increased to 4.03–1063.87). Also, the number of observations for this variable was lower (n = 83), and the goodness of fit decreased (Hosmer–Lemeshow *p* = 0.08). For these reasons, it was not maintained in the model.

The associations were consistent at Cox regression, in which the following variables were associated with the time to event: neurological impairment by computed tomography (HR = 3.69, 95% CI 1.32–10.35; *p* = 0.01), CSF yeast count-log (HR = 1.37, 95% CI 1 1.10–1.70, *p* = 0.004) and age in years (HR = 1.06, 95% CI 1.01–1.10, *p* = 0.01) and systemic arterial hypertension but with lower precision (HR = 2.56, 95% CI 0.88–7.47, *p* = 0.08).

**Fig. 3:**
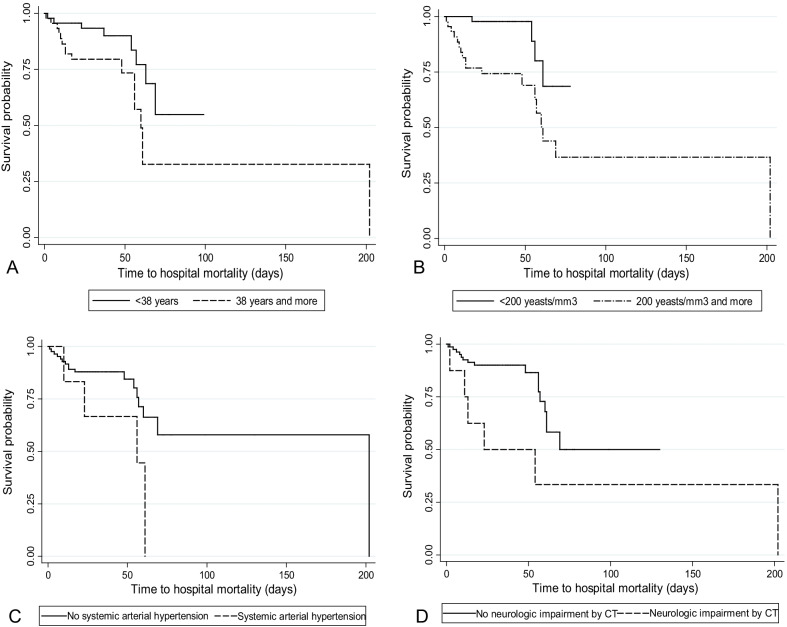
Survival curves until the hospital mortality, according to the variables selected for the multiple analysis model. **A** Age, **B** CSF yeast count and **C** Systemic arterial hypertension and **D** Neurologic impairment by computed tomography (CT)

For the representation with Kaplan–Meier curves, the quantitative variables were categorized according to the respective medians (i. e. 38 years for age and 200 yeasts/mm^3^ for CSF yeast count). Since the curves did not intersect, it was assumed that the proportionality of the risks was maintained throughout the time of hospitalization (Fig. 3). A similar result was observed in survival curves adjusted (see Additional file 1: Fig. S2).

### Secondary analysis—predictors of the composite outcome

The variables that presented statistically significant difference regarding the composite outcome were: age, Glasgow scale, headache, cerebral toxoplasmosis, systemic arterial hypertension, pneumonia, culture of *Cryptococcus* spp. from bloodstream, culture of *Cryptococcus spp.* from extra neural sites, CSF yeasts count, urea level and cerebral edema and ventricular dilation by computed tomography (Table 3).

**Table 3:** Univariable analysis of prognostic variables related to hospital mortality and/or ICU transfer during hospitalization in 96 patients with cryptococcal meningitis at Emilio Ribas Institute of Infectious Diseases, São Paulo, Brazil

Variables	All	Death or ICU transfer (n = 37)	Survivor or no ICU transfer (n = 56)	p*
Age (years)-average (IQR)	38.7 (29–46)	41.8 (30.5–51)	36.3 (28–44.5)	0.06
Glasgow coma scale (IQR)	14 (15)	14 (14–15)	15 (15–15)	< 0.001
Signs and symptoms at admission				
Headache	77/90 (85.56%)	25/34 (73.53%)	52/56 (92.86%)	0.03
Mental confusion	19/90 (21.11%)	11/34 (32.35%)	8/56 (14.29%)	0.06
Seizure	12/90 (13.33%)	7/34 (20.59%)	5/56 (8.93%)	0.2
Nausea	28/90 (31.11%)	7/34 (20.59%)	21/56 (37.5%)	0.11
Cough	2/90 (2.22%)	2/34 (5.88%)	0/56 (0%)	0.14
Neck stiffness	3/90 (3.33%)	3/34 (8.82%)	0/56 (0%)	0.05
Storpor	4/90 (4.44%)	3/34 (8.82%)	1/56 (1.79%)	0.15
Clinical-laboratory data				
Cerebral toxoplasmosis	9/90 (10%)	7/34 (20.59%)	2/56 (3.57%)	0.02
Systemic arterial hypertension	6/90 (6.67%)	5/34 (14.71%)	1/56 (1.79%)	0.03
Lymphoma	3/90 (3.33%)	3/34 (8.82%)	0/56 (0%)	0.05
Pneumonia	11/90 (12.22%)	9/34 (26.47%)	2/56 (3.57%)	0.002
Culture of *Cryptococcus* spp. from bloodstream	18/89 (20.22%)	12/34 (35.29%)	6/55 (10.91%)	0.007
Culture of *Cryptococcus spp.* from extra neural sites	20/89 (22.47%)	12/34 (35.29%)	8/55 (14.55%)	0.03
CSF yeasts count (yeasts/mm^3^) average (IQR)	749 (6–665)	1531 (73–2240)	275 (2.5–330)	0.002
Urea level (mg/dL) average (IQR)	33.41 (21–40)	40.91 (22–48)	28.46 (21–34)	0.04
HIV viral load average (IQR)	242,556 (1824–332,156)	280,197 (7912–3,826,251)	221,890 (495 -311,000)	0.18
Regular antiretroviral therapy	17/90 (18.89%)	3/34 (8.82%)	14/56 (25%)	0.09
*C. neoformans* VNI	77/96 (80.21%)	27/37 (72.97%)	48/56 (85.71%)	0.18
AMB-FCZ therapy	42/89 (47.19%)	12/34 (35.29%)	30/55 (54.55%)	0.09
FCZ MIC (mg/L) average (IQR)	9 (1–16)	8 (1–8)	10 (1–16)	0.20
FCZ MIC ≥ 16 mg/L	27/96 (28.13%)	7/37 (18.92%)	20/56 (35.71%)	0.10
Neuroimaging-computed tomography				
Cerebral edema	4/90 (4.44%)	4/34 (11.76%)	0/56 (0%)	0.02
Ventricular dilation	4/90 (4.44%)	4/34 (11.76%)	0/56 (0%)	0.02
No alterations	42/90 (46.67%)	11/34 (32.35%)	31/56 (55.36%)	0.05
Neuroimaging-magnetic resonance imaging				
Pseudocysts	4/90 (4.44%)	3/34 (8.82%)	1/56 (1.79%)	0.15

^*^p ≤ 0.2; IQR, interquartile range 25–75%; CSF, cerebrospinal fluid; AMB, amphotericin B; FCZ, fluconazole; MIC, minimum inhibitory concentration

At the multiple model, the CSF yeast count-log (OR = 1.37, 95% CI 1.11–1.71, *p* = 0.004), culture of *Cryptococcus* spp. from bloodstream (OR = 3.66, 95% CI 1.08–12.30, *p* = 0.04) and cerebral toxoplasmosis (OR = 12.53, 95% CI 2.04–76.93, *p* = 0.006) were associated with this outcome. This model showed the area under the ROC curve of 76.2% (95% CI 0.70–0.90). The accuracy was 78.65%, with a sensitivity of 61.7% and specificity of 89.1% (positive predictive value 77.8%, negative predictive value 79.0%). This model showed the goodness of fit with *p* = 0.71 and *p* = 0.74 to the Pearson and the Hosmer–Lemeshow test, respectively.

When the extreme and the influential observations were excluded, the CSF yeast count-log remained associated with the composite outcome (Additional file 1: Fig. S3, Table S3 and S4).

**Fig. 4:**
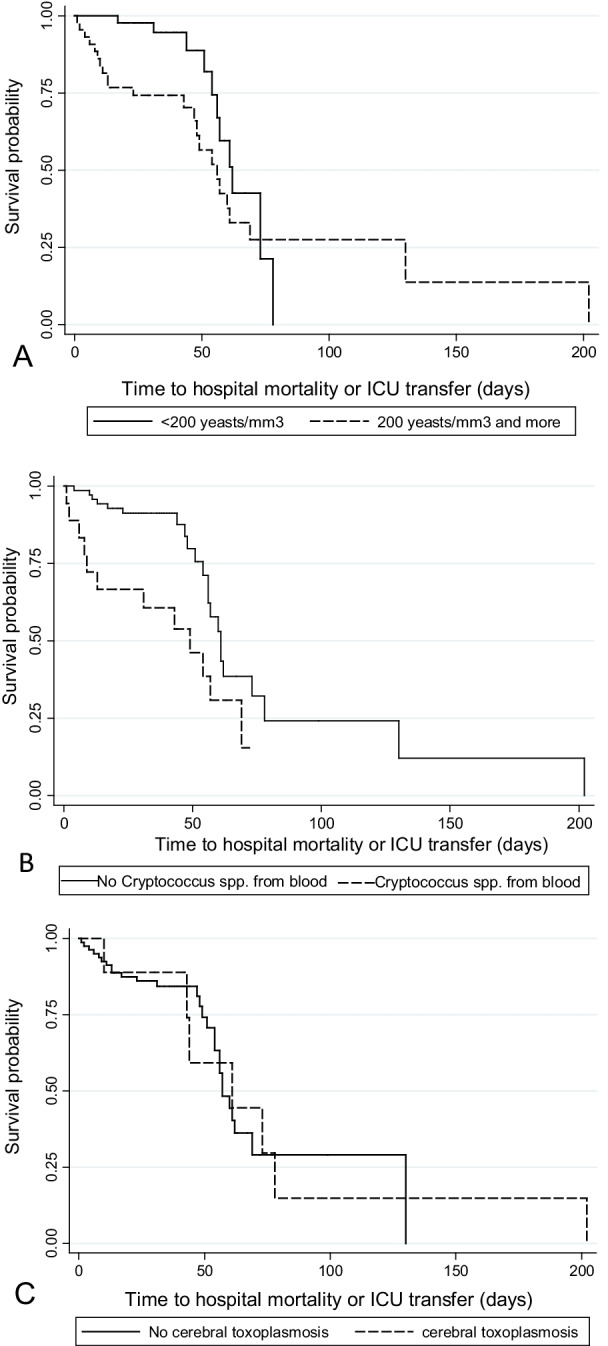
Survival curves until the occurrence of the composed outcome (hospital mortality and/orICU transfer), according to the variables selected for the multiple analysis model. **A** CSF yeasts count, **B** culture of *Cryptococcus* spp. from bloodstream and **C** concomitant cerebral toxoplasmosis

The increase of one unit of CSF yeasts count-log was associated with the acceleration of 14% to the occurrence of one of the events, observed in the Cox regression model (Hazard ratio [HR] = 1.14, 95% CI 0.99–1.33 *p* = 0.07). The culture of *Cryptococcus* spp. exhibited an HR of 2.1 in the same model (95% CI 0.98–4.53, *p* = 0.05). Diagnosis of cerebral toxoplasmosis showed an HR of 1.44 (95% CI 0.546–3.82, *p* = 0.46).The patients who had CSF yeast count ≥ 200 yeasts/mm^3^, culture of *Cryptococcus* spp. from bloodstream or cerebral toxoplasmosis presented lower survival time related to the occurrence of one of the events of the composite outcome (Fig. 4).

## Discussion

We studied a cohort of patients with cryptococcal meningitis, which were predominantly from PLWHA and, as in other studies, the majority were males, also reflecting the population most affected by the AIDS epidemic [51–57]. The in-hospital mortality observed was 26.9% and, according to other Brazilian studies, this rate may vary from 25 to 74%, especially in the first weeks of therapy [4, 55, 58–62].

In Brazil, the etiologic agents of cryptococcal meningitis are, in their vast majority, *C. neoformans s.s.*, also confirmed in this series of patients [4, 11, 55, 62–68]. Of the 3 cases of *C. deuterogattii* infection, 2 were negative HIV, and among them, one of them was diagnosed with primary immunodeficiency. About the gravity of these patients, no association was observed between the species and the outcomes. No influence was observed at the analysis without the *C. deuterogatti* isolates (data no shown). The presence of cryptococcomas is most usual in *C. deuterogattii* infections and it can cause additional neurological damage, impair the antifungal drugs access to infection sites and need prolonged therapy [69–74]. The cryptococcomas were described at 5 patients, 2 *C. neoformans* VNI, 2 *C. neoformans* VNII and 1 case who resulted in the only death with *C. deuterogattii* isolation. Among the 25 cases of in-hospital death, the majority presented *C. neoformans* VNI (17), followed by 7 *C. neoformans* VNII and 1 *C. deuterogattii* isolates. It is known that *C. deuterogattii* is the second specie most frequent, and it shows greater virulence when compared to *C. neoformans*, which could justify the individuals without apparent immunosuppression can be infected by this specie. However, other factors may be associated with and interfere with the immune response of patients, such as age and the presence of other comorbidities [63, 75–77]. As described in studies conducted in Southern Brazil, *C. neoformans* VNI overtake the others in this study, and among *C. gattii* complex, *C. deuterogattii* was exclusive [78–82].

The multiple analysis revealed important variables potentially available in the first week of hospitalization, including age, CSF yeast count, systemic arterial hypertension and neurological impairment identified by CT. The survival analysis indicated that these variables were consistent predictors of time to in-hospital mortality, even when adjusted. The Cox regression indicated that these variables maintained the associations, even after the exclusion of influential observations and outliers. In the secondary analysis, CSF yeast count, culture of *Cryptococcus* spp. from the bloodstream and cerebral toxoplasmosis were associated with the composed outcome. All these associations were consistent in the survival models.

The age was significantly associated with in-hospital death, and this result was also observed at the multiple analysis confirming that age can be a risk factor for death in these patients [52, 74, 83, 84]. A study performed in Taiwan observed that age > 60 years was a predictor of poor prognosis [52]. Another research with cryptococcal meningitis patients showed age > 50 years independently associated with death within two weeks [22]. Therefore, our study is consistent with these associations and we also suggest that age exhibited a biological gradient since the increase in years was progressively associated with the odds of death.

Fungal burden has been associated with therapeutic failure and death [4, 22, 43, 85–88]. However, fungal burden is formally evaluated with quantitative CSF cultures [22] but this methodology is usually unavailable in routine clinical practice of low and middle-income countries. In the present study, quantitative CSF yeast counts performed by direct microscopic exam was associated with mortality in multiple analysis by logistic regression. This association was suggested in the univariable analysis of a previous study performed at our institution, which include 46 HIV-infected patients with cryptococcal meningitis [88]. Thus, our results extend the value of quantitative CSF yeast counts performed by direct microscopic exam.

The presence of a high number of *Cryptococcus* spp. yeasts at CSF contribute to increase intracranial pressure, it being a mechanism proposed to the mechanical obstruction of CSF flow [89, 90]. Probably thick capsules and giant cells also contribute to increased intracranial pressure [91, 92]. Although there was no difference between increased intracranial pressure and death in this study, there was a trend of a greater number of cases of this variable related to death, as reported in the literature [7, 93–95]. Probably due to the sample size, it was not possible to estimate this association with precision. Furthermore, aggressive control of measures to control the increased intracranial pressure may have masked the effect of this variable on mortality.

The presence of *Cryptococcus* spp. antigen in CSF was not associated with outcomes in a prior study, suggesting that serum or CSF antigen dosage cannot be considered a predictive factor for failure to treat or relapse of cryptococcosis in this specific hospitalized population [7]. However, the dissemination of cryptococcosis, identified by the culture of *Cryptococcus* spp. from extra neural sites, was associated with an increased hazard of the composite outcome. This is consistent with previous reports in which the isolation of *Cryptococcus* spp. from the bloodstream was and predictor of poor clinical outcome [83, 94, 96–99].

The CD4 < 200 cells/mm^3^ was not associated with the outcomes of our study. However, it is known that when a variable presents low variability or values predominantly reduced, as in this case, the capacity of discrimination is reduced, which could explain the lack of association of this variable with the outcomes.

World Health Organization recommends screening all PLWHA who have a CD4 < 200 cells/mm^3^ for cryptococcal antigen to identify those patients who could benefit from preemptive fluconazole treatment prior to the onset of meningitis [7, 100]. Nevertheless, where cryptococcal antigen testing is not available, FCZ primary prophylaxis should be used in some epidemiological settings [7, 96, 100].

All patients were treated with AMBd during the therapy induction and the combined therapy was administered in almost all patients. According to recommendations, AMB-combination is the most appropriate therapy for cryptococcal meningitis [7, 26]. Due to the partial availability of 5FC at the hospital, this drug was given to almost half of the patients. In this study, in contrast to current recommendations [7], the use of 5FC or FCZ as a second drug was not associated with the outcomes (in-hospital mortality: 5FC *p* = 1 and FCZ  *p* = 0.6; composite outcome: 5FC *p* = 0.83 and FCZ *p* = 0.09). This result can indicate multiples intervening factors in this retrospective observational study. Similarly, antifungal susceptibility was not associated with the outcomes. Currently, the determination of AMB-MIC and FCZ-MIC are not recommended at the hospital routine, since the correlation between these results and the clinical response remains uncertain. Also, the determination of clinical breakpoints for *Cryptococcus* spp. remains non-existent due to the absence of studies correlating MIC or other susceptibility measures with the clinical prognosis [28, 29, 42, 47, 83, 101, 102]. All strains presented FCZ-heteroresistant clones however, even with high values (53.1% of strains with LHF ≥ 32 mg/L) the LHF was not associated with the severity of cryptococcal meningitis. In a previous study, LHF clones were obtained from *Cryptococcus neoformans* strains of the first episode of no-AIDS patient and from another patient with relapses of cryptococcosis. The LHF was similar to both [33]. On the other hand, a study showed distinct LHF at FCZ-resistant and susceptible strains and higher FCZ-MIC at respective heteroresistant clones [36]. Likewise, the fungicidal activity of AMB by Time-kill methodology showed no association with outcomes. Similar result was previously described [3]. On the other hand, a study reported an association of AMB-tolerant strains and mortality [41, 42]. Possibly the sample size can be responsible for the absence of this association in our study.

The sample size was a limiting factor that may have affected the precision of estimates, for instance, at the 95% CI of systemic arterial hypertension and that of the neurological impairment by CT, that it was wide. Despite this, associations were consistent among the models evaluated including those with binary and time-to-event outcomes. All associations with the outcomes were maintained in all models, like OR and OR adjusted and the survival models. These observations were maintained even when the extreme and influential observations were excluded and, therefore, these findings indicate these predictors are associated with the frequency and the velocity of the outcomes. Moreover, the associations were coherent with the expected according to the literature. For this reason, it is plausible that the associations observed could be extrapolated to other populations.

## Conclusion

We identified clinical and laboratorial factors observed in the first days of hospitalization able to predict the prognosis of cryptococcosis in a predominantly PLWHA. The associations remained consistent when evaluated in survival analyses. CSF yeast count was a consistent predictor of in- hospital mortality and severity and the methodology used in this study can be implemented in low and middle-income settings.

## Supplementary Information

**Additional file 1. SUPPLEMENTARY DATA Figure S1**- Delta-deviance and leverage of observations generating the multiple model using the variables age, CSF yeast count-log, systemic arterial hypertension and neurological impairment by computed tomography associated with hospital mortality. **Figure S2**- Survival curves until the in-hospital mortality, according to the variables selected for the multiple analysis model adjusted. **Figure S3** - Delta-deviance and leverage of observations generating the multiple model using the CSF yeast count-log, positive blood culture for Cryptococcus spp. and cerebral toxoplasmosis, associated with composed outcome. **Table S1**- **Multiple** analysis by logistic regression of variables associated to in-hospital mortality, without the influential observations. **Table S2** - Multiple analysis by logistic regression of variables associated to in-hospital mortality, without the outliers. **Table S3** – Multiple analysis by logistic regression of variables associated to composed outcome, without the influential observations. **Table S4** - Multiple analysis by logistic regression of variables associated to composed outcome, without the outliers.

## Data Availability

The datasets used and analyzed during the current study are part of the patient's medical records. They are available from the corresponding author on reasonable request.
