# Supplementary material for: Early clinical and microbiological predictors of outcome in hospitalized patients with cryptococcal meningitis
Source: BMC Infect Dis. 2022 Feb 9;22:138. doi: 10.1186/s12879-022-07118-7 (PMC8830130; doi:10.1186/s12879-022-07118-7)
Supplement: Supplementary file 1 — Additional file 1. SUPPLEMENTARY DATA Figure S1- Delta-deviance and leverage of observations generating the multiple model using the variables age, CSF yeast count-log, systemic arterial hypertension and neurological impairment by computed tomography associated with hospital mortality. Figure S2- Survival curves until the in-hospital mortality, according to the variables selected for the multiple analysis model adjusted. Figure S3 - Delta-deviance and leverage of observations generating the multiple model using the CSF yeast count-log, positive blood culture for Cryptococcus spp. and cerebral toxoplasmosis, associated with composed outcome. Table S1- Multiple analysis by logistic regression of variables associated to in-hospital mortality, without the influential observations. Table S2 - Multiple analysis by logistic regression of variables associated to in-hospital mortality, without the outliers. Table S3 – Multiple analysis by logistic regression of variables associated to composed outcome, without the influential observations. Table S4 - Multiple analysis by logistic regression of variables associated to composed outcome, without the outliers. [file 12879_2022_7118_MOESM1_ESM.docx]

**SUPPLEMENTARY DATA**

During the evaluation of the multiple model, it was observed that there were 3 influential observations (with delta-deviance higher than the delta-deviance cutoff point of 4) and 6 outliers (observations with a leverage greater than 3 times the average of the sample) and after exclusion of those observations the associations found maintained their associations (Table S1 and Table S2).

Figure S1- Delta-deviance and leverage of observations generating the multiple model using the variables age, CSF yeast count-log, systemic arterial hypertension and neurological impairment by computed tomography associated with hospital mortality

Figure S2- Survival curves until the in-hospital mortality, according to the variables selected for the multiple analysis model adjusted. A1) Age adjusted by systemic arterial hypertension, A2) Age adjusted by CSF yeast count, A3) age adjusted by neurologic impairment by computed tomography, B1) CSF yeast count adjusted by systemic arterial hypertension, B2) CSF yeast count adjusted by neurologic impairment by computed tomography, B3) CSF yeast count adjusted by age, C1) Systemic arterial hypertension adjusted by CSF yeast count, C2) Systemic arterial hypertension by age, C3) Systemic arterial hypertension adjusted by neurologic impairment by computed tomography, D1) Neurologic impairment by computed tomography adjusted by age, D2) Neurologic impairment computed tomography adjusted by CSF yeast count, D3) Neurologic impairment computed tomography adjusted by systemic arterial hypertension

A1 A2

A3

B1 B2 B3

C1 C2

C3

D1 D2

D3

Figure S3 - Delta-deviance and leverage of observations generating the multiple model using the CSF yeast count-log, positive blood culture for *Cryptococcus* spp. and cerebral toxoplasmosis, associated with composed outcome (in-hospital mortality or intensive care unit transfer).

Table S1- Multiple analysis by logistic regression of variables associated to in-hospital mortality, without the influential observations (with delta-deviance higher than the cutoff point of 4)

| **Variable** | **Odds Ratio** | **Confidence Interval 95%** | ***p*** |
| --- | --- | --- | --- |
| Age (years) | 1.13 | 1.04-1.23 | 0.005 |
| CSF yeast count-log | 2.68 | 1.46-4.93 | 0.001 |
| Systemic arterial hypertension | 137.50 | 3.71-5098.80 | 0.008 |
| Neurological impairment by computed tomography | 646.52 | 2.55-164056.2 | 0.02 |

CSF, cerebrospinal fluid

Table S2 - Multiple analysis by logistic regression of variables associated to in-hospital mortality, without the outliers (observations with a leverage greater than 3 times the average of the sample = 0.1694058)

| **Variable** | **Odds Ratio** | **Confidence Interval 95%** | ***p*** |
| --- | --- | --- | --- |
| Age (years) | 1.07 | 1.00-1.14 | 0.04 |
| CSF yeast count-log | 2.21 | 1.31-3.71 | 0.003 |
| Systemic arterial hypertension | 1 (omitted) |  |  |
| Neurological impairment by computed tomography | 6.00 | 0.53-67.82 | 0.14 |

CSF, cerebrospinal fluid

Table S3 – Multiple analysis by logistic regression of variables associated to composed outcome (in-hospital mortality or intensive care unit transfer), without the influential observations (with delta-deviance higher than cutoff point of 4)

| **Variable** | **Odds Ratio** | **Confidence Interval 95%** | ***p*** |
| --- | --- | --- | --- |
| CSF yeast count-log | 1.42 | 1.13-1.79 | 0.003 |
| culture of *Cryptococcus* spp. from bloodstream | 3.56 | 1.04-12.15 | 0.042 |
| cerebral toxoplasmosis | 31.63 | 3.01-332.24 | 0.004 |

CSF, cerebrospinal fluid

Table S4 - Multiple analysis by logistic regression of variables associated to composed outcome (in-hospital mortality or intensive care unit transfer), without the outliers (observations with a leverage greater than 3 times the average of the sample=0.251226)

| **Variable** | **Odds Ratio** | **Confidence Interval 95%** | ***p*** |
| --- | --- | --- | --- |
| CSF yeast count-log | 1.57 | 1.177-2.091 | 0.002 |
| culture of *Cryptococcus* spp. from bloodstream | 4.11 | 1.15-14.77 | 0.030 |
| cerebral toxoplasmosis | 19.89 | 2.64-149.91 | 0.004 |

CSF, cerebrospinal fluid
